# Prediction of the photodynamic therapy effect using digital breast phantoms from patients with breast cancer via Monte Carlo simulations

**DOI:** 10.1117/1.JBO.30.S3.S34110

**Published:** 2025-09-23

**Authors:** Yugo Minegishi, Yasutomo Nomura

**Affiliations:** Maebashi Institute of Technology, Graduate School of Engineering, Gunma, Japan

**Keywords:** photodynamic therapy, breast cancer, near-infrared, digital breast phantom, Monte Carlo simulation, upconversion

## Abstract

**Significance:**

Photodynamic therapy (PDT) agents activated by near-infrared (NIR) light have demonstrated effectiveness in animal studies. However, clinical trials in humans are lacking due to biocompatibility concerns. We evaluate the feasibility of NIR-PDT using newly developed upconversion nanoparticles–quantum dots–Rose Bengal (UCQRs) through Monte Carlo simulations.

**Aim:**

Surgery, the primary treatment mode for breast cancer, often reduces the quality of life due to scarring, necessitating a less invasive alternative. Herein, we propose an NIR-PDT approach using UCQRs to treat patients with early-stage breast cancer. The treatment can be performed on patients in the prone position using light irradiation alone, significantly reducing the burden on patients. In NIR-PDT using UCQR, a treatment depth of 3 to 4 cm can be expected based on the penetration depth of the 808-nm excitation light.

**Approach:**

We created 150 digital breast phantoms by reconstructing breast slice images from breast computed tomography scans. These phantoms were classified by breast density and tumor depth, and simulations were performed on representative models. The therapeutic effect of NIR-PDT was assessed based on the amount of singlet oxygen generated, calculated from the fluence in the tumor voxels.

**Results:**

The simulations indicated that tumor depth had a greater impact on the therapeutic outcomes compared with breast contour or structure. In all phantoms where tumors with a 7-mm diameter were embedded at depths of 15 to 25 mm, the generated singlet oxygen exceeded the cell death threshold across all tumor voxels. Shallow tumors between 15 and 20 mm can be treated with 15 or fewer irradiations, whereas deep tumors between 20 and 25 mm are estimated to require up to 45 irradiations.

**Conclusions:**

This virtual clinical trial using 150 digital phantoms suggests that NIR-PDT with UCQRs offers a promising, minimally invasive alternative for treating breast cancer.

## 1 Introduction

Breast cancer, one of the most common cancers in women, affects ∼7% of women before the age of 40 years. The quality of life (QOL) of these patients after treatment is a critical concern. Although radiation therapy, medication, and adjuvant therapies are sometimes employed, surgery remains the primary treatment for most breast cancer cases. However, surgical treatment can significantly impact QOL due to changes in breast shape.^1^ Breast-conserving surgery and reconstruction can help preserve the appearance of the breast, but it is challenging to completely eliminate surgical scars or correct left–right asymmetry.^2^ Therefore, alternative treatment methods that reduce the physical and emotional burden of surgery are urgently needed.

Phototherapy technologies such as photoimmunotherapy (PIT), photothermal therapy (PTT), and photodynamic therapy (PDT) are less invasive treatment alternatives for breast cancer. In PIT, the host’s antitumor immune response is activated by injecting photoavailable agents, such as IR-700, which target tumor cells and are activated by light at 690 nm.^3^ However, the limited tissue penetration of visible light poses a challenge in delivering sufficient energy to deeper breast tumors. PTT treats tumors by generating heat via light irradiation of accumulated photothermal agents within tumor tissues,^4^ but it carries the inherent risk of thermal damage to surrounding healthy tissues.^5^ Furthermore, PTT alone may not completely eradicate the tumor and is therefore often considered more suitable as an adjunctive therapy.^6^ In conventional PDT, photosensitizers within cancer cells absorb visible light and produce reactive oxygen species, which in turn destroy tumor cells.^7^ However, as with PIT, the limited penetration of visible light limits the application of conventional PDT. Interstitial PDT greatly enhances the treatment depth by delivering light directly into the tumor through the insertion of a laser fiber using a needle or catheter.^8^ However, issues such as pain during or after treatment and unintentional bleeding due to fiber insertion have been reported.^9^ Recent advances in nanotechnology have led to the development of PDT in the near-infrared (NIR) region (NIR-PDT).^10^ NIR light penetrates deeper into biological tissues than visible light due to reduced scattering and absorption,^11^ overcoming the issue of limited light penetration that is characteristic of conventional PDT. Furthermore, recent studies have shown that diagnostic methods in the prone position reduce pain caused by breast compression during breast cancer screening.^12^^,^^13^ NIR-PDT allows tumor irradiation while the patient remains in a comfortable prone position, making it a promising treatment for breast cancer.^14^ In many cases of NIR-PDT, nanoparticles are used due to their advantages such as targeted delivery, increased loading capacity, and multifunctionality.^15^ Currently, metallic nanoparticles (NPs), ceramic NPs, carbon-based NPs, and lanthanide-based NPs have been developed. In particular, lanthanide-based NPs are expected to be applied to NIR-PDT for deep tumors due to their upconversion properties, which convert NIR light into visible light. The upconversion nanoparticles–quantum dots–Rose Bengal (UCQRs) system as the lanthanide-based NPs for the PDT in the NIR region, developed by Song et al., efficiently absorbs excitation energy at 808 nm through the quantum dots, which is then converted into higher-energy visible light by the upconversion nanoparticles (UCNPs), effectively activating the photosensitizer.^16^ The most distinctive feature of UCQR compared with other lanthanide-based NPs is the use of quantum dots to enhance the absorption of 808-nm excitation light. The strategy of NIR-PDT using upconversion nanoparticles has been challenged by the extremely low absorption efficiency of excitation light due to the very small absorption cross-section of UCNPs.^16^ Therefore, in UCQR, quantum dots with strong absorption at 808 nm are placed around the UCNPs, significantly improving the absorption efficiency of excitation light. To our knowledge, there are few studies reporting the therapeutic effects of NIR-PDT using quantum dots in *in vivo* experiments.^17^ In addition, UCQRs specifically accumulate in tumor cells via the enhanced permeability and retention (EPR) effect, selectively releasing singlet oxygen in the tumor tissues to effectively kill cancer cells while minimizing the damage to the surrounding healthy tissues. Due to these benefits, mouse experiments using NIR-PDT with UCQRs have shown significant tumor volume reduction.^16^ On the other hand, previous *in vivo* studies have reported tissue penetration of ∼3 to 4 cm for 808-nm light in lean bovine tissue samples and human brain tissue.^18^^,^^19^ As human breast tissue has lower absorption and scattering coefficients than lean bovine tissue and brain tissue, it is expected that the light can penetrate deeper.^20^ Therefore, the treatment depth of NIR-PDT using UCQR is anticipated to be at least 3 to 4 cm or greater. However, many NIR-PDT agents, including UCQRs, have not been applied to the human body due to the limited information regarding the biocompatibility of the rare earth elements used in UCNPs.^16^^,^^21^

In our previous study, we reported that NIR-PDT using UCQRs can effectively treat early-stage breast cancer, as demonstrated through Monte Carlo (MC) modeling.^22^ We accurately reconstructed the contour and internal structure of an uncompressed breast in the prone position using an available digital dataset.^23^ We then used this simulation to estimate that spherical tumors with diameters ranging from 5 to 9 mm could be completely treated at a typical depth of 15 to 25 mm. However, the influence of individual variations in breast contour and internal mammary structure on treatment efficacy was not explored.

In this study, which includes typical breast types, we focus on uncompressed computational breast phantoms generated from 150 clinical breast images. First, a cohort of 150 phantoms with varying breast contours and internal glandular structures was categorized according to breast density. Subsequently, a small spherical region, representing early-stage cancer, was embedded at a common site within each phantom based on the three-dimensional (3D) structure of the mammary gland. Each embedded tumor was assumed to be guided by either magnetic resonance imaging (MRI) or fluorescence molecular imaging, as reported recently.^22^^,^^24^ Next, the therapeutic effect of NIR-PDT on the embedded tumor was assessed using MC simulations. Light propagation within each tumor-bearing phantom was then calculated through the simulation. Based on the fluence accumulated within the tumor voxels, the quantity of singlet oxygen generated by UCQRs was estimated. The therapeutic outcome for the tumor voxels was determined by comparing the generated singlet oxygen levels with the apoptotic threshold required for tumor cell death. The simulation findings indicated that UCQRs within a 7-mm-diameter tumor, located at a depth of 15 to 25 mm, could produce sufficient singlet oxygen to induce apoptosis after multiple light exposures. Therefore, this virtual clinical trial using 150 digital breast phantoms suggests that NIR-PDT combined with UCQRs represents a promising next-generation treatment strategy for early-stage breast cancer.

## 2 Methods

### 2.1 Reconstruction of the 3D Digital Breast Phantoms

**Fig. 1:**
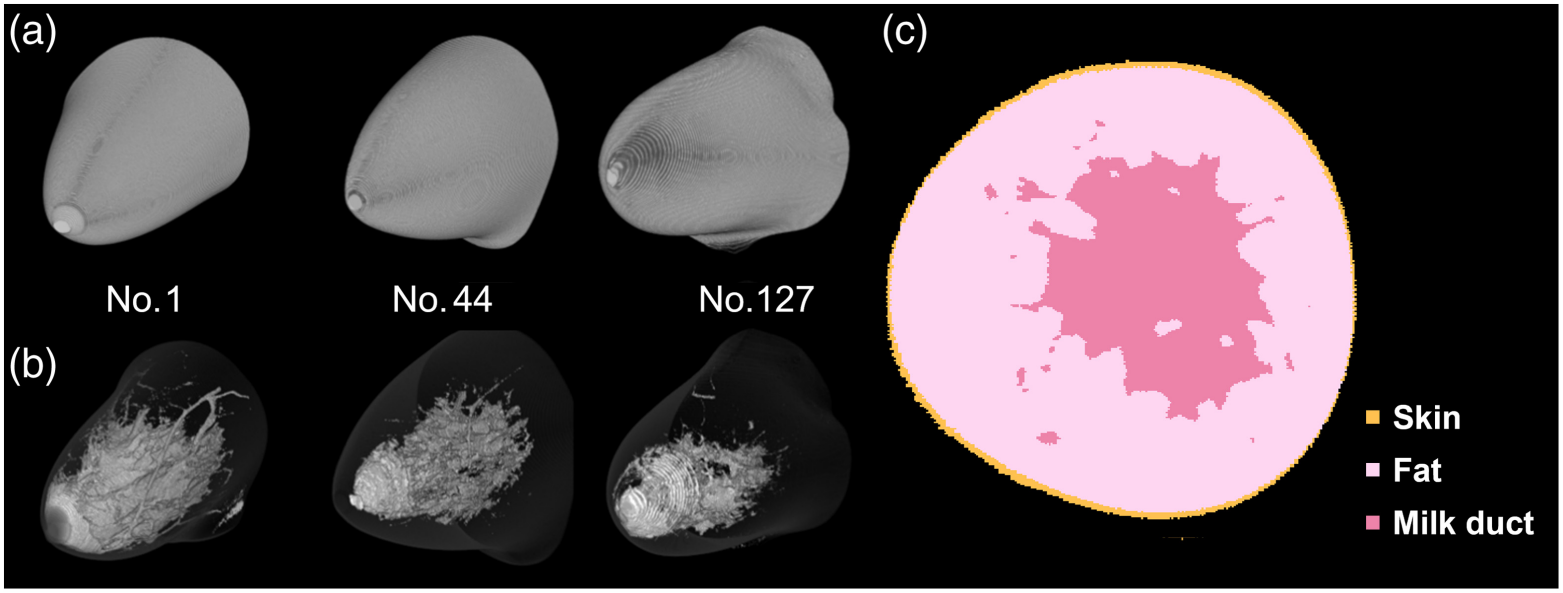
Typical images of the breast contours (a), internal structures of the mammary gland (b) in the phantoms, and a coronal slice (c). In panels (a) and (b), the upper and lower images are derived from the same patients: nos. 1, 44, and 127. (c) Slice no. 330 of the phantom from patient no. 9.

Several recently available breast cancer datasets were used to create the 3D breast phantoms for the MC simulation in this study. The Digital Database for Screening Mammography and the chest X-ray database “ChestX-ray8” have been published as breast slice images captured by digital mammography or X-ray.^25^^,^^26^ However, each breast consists of a small number of slices in these datasets, making it difficult to reconstruct a realistic 3D breast phantom. Conversely, computed tomography (CT) images of the whole body and female breasts were obtained from the Visible Human Project (VHP),^27^ the Chinese Visible Human (CVH) project,^28^ and research on breast computed tomography (BreastCT) by Sarno et al.^23^ The VHP dataset includes ∼500 slice images of the bilateral breasts in the supine position. Therefore, a realistic 3D breast phantom can be reconstructed from this dataset. However, investigating the effects of individual differences in breast shape and mammary structure was challenging because the data were obtained from a single person. The CVH project has released 400 whole-body slice images from CT and MRI, but the data are currently unavailable. Sarno et al. released a dataset of breast images acquired in the prone position using BreastCT for in silico studies. This dataset comprises 150 breast phantoms, each consisting of ∼500 slice images,^23^ which efficiently replicated individual variations in breast contours and internal glandular structures. In addition, it is straightforward to set the optical properties for each tissue, as each slice image is labeled with three tissue types: skin, fat, and milk ducts, excluding tumor regions.^23^ Thus, in this study, 150 breast phantoms were reconstructed from the dataset provided by Sarno et al. During phantom reconstruction, the pixel sizes of the 150 breast images in the dataset were standardized to 0.33 mm to simplify the simulation. The pixel size was chosen based on the original breast images in the dataset, which ranged from 0.19 to 0.43 mm.^23^ Therefore, the pixel size used in this study is based on the spatial resolution of breast CT and is likely sufficiently small to reproduce breast structures accurately. In addition, it is nearly comparable in size to other MC simulation studies.^29^^,^^30^ Then, the images in the dataset, originally composed of 512×512  pixels, were resized to 500×500  pixels by removing the background-colored pixels at the edges. A 3D breast phantom was reconstructed by stacking these images with a thickness of 0.33 mm. Therefore, each digital breast phantom, represented as a voxel-based model, consisted of 500×500×500  voxels, with each voxel measuring $0.33\times 0.33\times 0.33\;{\mathrm{mm}}^{3}$ in volume. Figure 1 presents examples of the contour (a) and the internal mammary gland structures (b) of the breast phantoms of patient nos. 1, 44, and 127, as well as a coronal slice image (C) corresponding to slice no. 330 from the phantom of patient no. 9. The phantom numbers referenced in the text align with those assigned by Sarno et al.^23^

### 2.2 Classification of the Breast Phantom and Criteria for Tumor Embedding

Because tumors were not labeled in the dataset images, they were embedded within the breast phantoms to simulate early-stage breast cancer in patients, which was represented by ductal carcinoma *in situ* (DCIS) in this study. DCIS remains confined to the milk ducts and does not invade the surrounding breast tissue.^31^ Therefore, tumors are less likely to occur in breasts with low breast density, defined as the ratio of duct volume to the combined volume of ducts and fat. Accordingly, breast phantoms were classified based on breast density, and appropriate phantoms representing breast cancer cases were selected. For classification, the Breast Imaging-Reporting and Data System (BI-RADS) was referenced,^32^ which classifies breasts based on both density and ductal distribution. However, as ductal scattering is challenging to evaluate, the classification in this study was based solely on breast density. Tumors were then embedded in the selected phantoms according to the following four criteria: (1) the tumor is spherical, with a diameter of 7 mm, which is a typical target for NIR-PDT;^22^ (2) tumor depth, defined as the distance from the tumor’s upper surface to the skin, is limited to 15 to 25 mm, representing the standard clinical range;^22^^,^^33^^,^^34^ (3) the tumor must be entirely located within the breast ducts; and (4) if multiple embedding sites are available, the location closest to the skin is selected. Previously, we investigated the tumor diameter and depth relevant to NIR-PDT in breast cancer. Moreover, as spherical tumors with diameters of 5 to 9 mm could be treated by NIR-PDT within the common breast cancer sites,^22^ the tumor diameter was set to 7 mm, which is the midpoint of the 5 to 9 mm range.

### 2.3 PDT Simulation

#### 2.3.1 Characteristics of the NIR-PDT agent

Nanotechnology research has shown that the diameter of nanoparticles influences their accumulation capacity in cancerous tissues.^35^ UCQRs possess the unique ability to specifically accumulate in tumors due to the EPR effect.^16^ The optimal particle diameter is determined by several factors, including permeability through the tumor neovasculature, clearance by the kidneys and liver, and blood circulation time. The particle diameter of UCQRs is ∼60 to 70 nm, which is small enough to permeate the blood vessel walls of neovascularized tumors but too large to pass through in the normal vasculature. In addition, intravenously injected UCQRs exhibit relatively long circulation times in the bloodstream while avoiding rapid renal clearance, enhancing their preferential accumulation in tumor tissues.

#### 2.3.2 Upconversion of the UCQRs

**Fig. 2:**
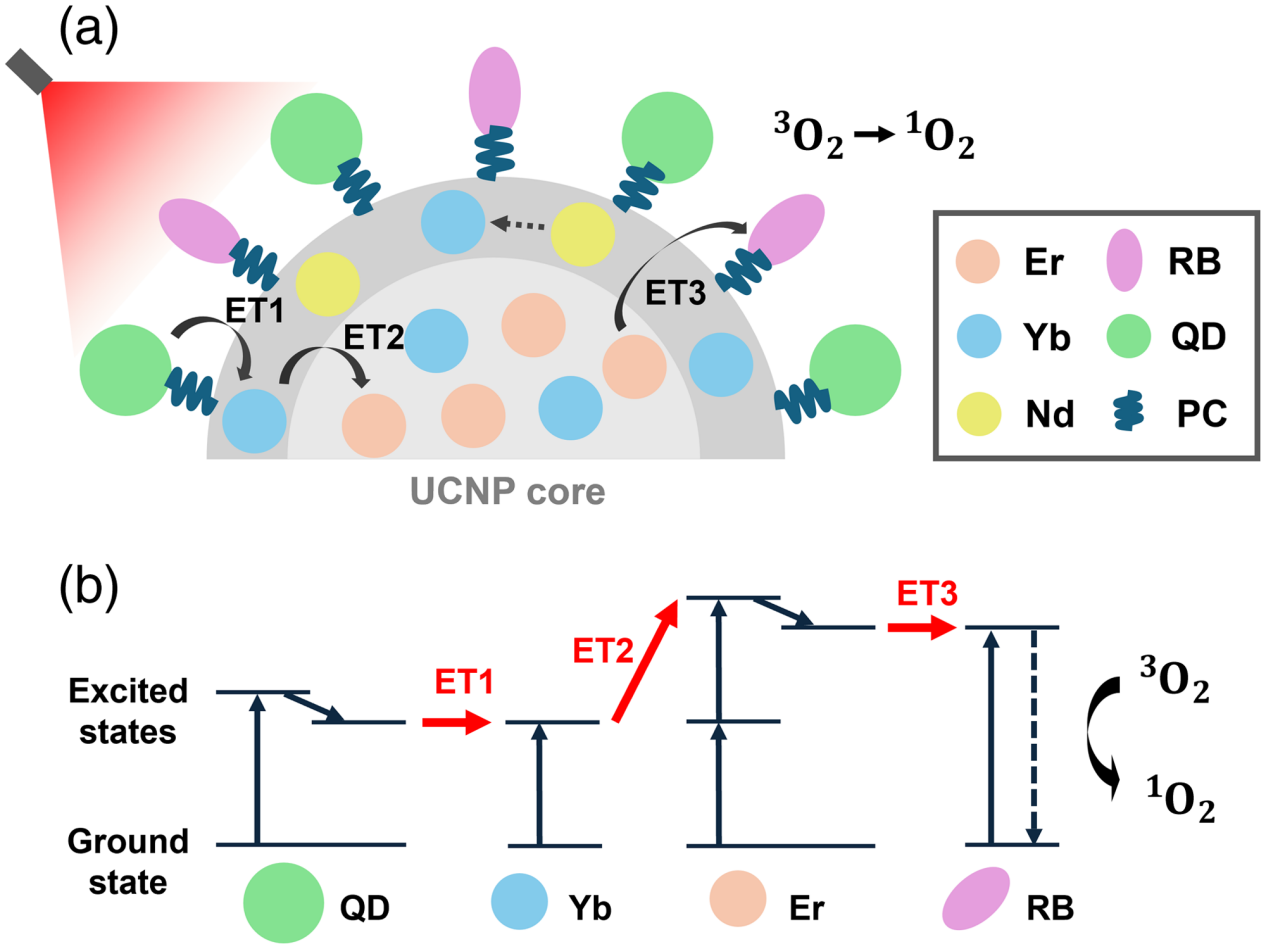
Schematic illustration of the UCQR-mediated singlet oxygen (O12) generation from triplet (O32), modified from Ref. 15. (a) Conceptual structure with functional components: Er, erbium; Yb, ytterbium; Nd, neodymium; RB, Rose Bengal; QD, quantum dots; PC, phosphatidylcholine. ET1 represents energy transfer from QD to Yb, ET2 from Yb to Er, and ET3 from Er to RB. The inner circle denotes the UCNP core. (b) Energy level diagram showing the ground and excited states. In ET2, upconversion occurs as Er is simultaneously excited by multiple photons.

The conceptual (a) and energy level (b) diagrams shown in Fig. 2 illustrate the structure and function of the UCQRs in generating singlet oxygen. In the initial step of singlet oxygen generation, the excitation energy absorbed by the quantum dot (QD) is transferred to ytterbium (Yb) ions (ET1). The QDs, arranged on the surface of the UCNP, efficiently absorb NIR light at 808 nm. In this system, the distance between the QDs and Yb ions within the UCNP is less than 10 nm, enabling resonance energy transfer (RET) to occur with high probability.^36^ In our previous study, we estimated the energy transfer efficiency (ETE) from the QDs to the Yb ions (ET1) to be 16.3%, based on the fluorescence lifetime of the QDs.^22^ The next step involves photon upconversion in the erbium (Er) ions within the UCNP, which occurs through the energy transfer from Yb to Er via multiphoton excitation (ET2). The UCNP core has an average diameter of 18 nm, which is close enough to allow RET from Yb to Er.^16^ During this energy transfer process, two or more low-energy photons are converted into a single high-energy photon. Notably, four energy transfer pathways exist from Yb to Er, resulting in Er fluorescence emission at four distinct wavelengths: 410, 540, 660, and 1540 nm. Previously, we estimated the transfer efficiencies for each pathway by comparing the photon energy at the four peak wavelengths relative to that at 410 nm.^16^^,^^22^ The transfer efficiency for 540-nm fluorescence—used to excite RB—was estimated at 50%.^22^ For Yb–Er upconversion to occur, the simultaneous absorption of two or more photons is required. However, accurately determining the likelihood of multiphoton excitation remains challenging. In this study, the probability of multiphoton excitation was assumed to range from 20% to 100% in 20% increments. Therefore, the ET2 efficiency was defined as the product of the 540-nm transfer efficiency (50%) and the assumed probability of multiphoton excitation (20% to 100%), resulting in a combined efficiency range of 10% to 50% in 10% increments. Here, Yb–Er upconversion has a nonlinear dependence under low-intensity excitation, and ET2 is affected by the excitation fluence.^37^ In this study, a constant ET2 was set at each voxel within the tumor, so the amount of singlet oxygen generated in tumors located deeper than the skin may have been overestimated, compared with tumors located shallower. However, it is difficult for us to experimentally investigate the excitation intensity-dependent ET2 in detail. Therefore, the dependence of ET2 on the excitation fluence at each voxel within the tumor was not considered in this study.

#### 2.3.3 Production of singlet oxygen

The final step involves energy transfer (ET3) from Er to RB, followed by the generation of singlet oxygen by RB molecules, which are arranged around the surface of the UCNP and serve as photosensitizers. The fluorescent intensity of Er within UCNPs containing RB was lower than that of UCNPs without RB, clearly indicating RET from Er to RB. Based on the fluorescence intensity ratio, the ET3 from Er to RB was set at 75%.^16^^,^^22^ Then, based on the estimates for ET1 to ET3, the overall ETE within UCQR was calculated as ETE = ET1 × ET2 × ET3. Depending on the variation in ET2, the ETE was set to 1.2%, 2.4%, 3.7%, 4.9%, and 6.1%, respectively.

The therapeutic effect of NIR-PDT using UCQRs on tumors was estimated based on the amount of singlet oxygen produced. This quantity was calculated as the PD, defined as the number of excitation photons absorbed by the photosensitizer.^38^ The photodynamic dose (PD) was then obtained using the following equation:

$$\frac{d{\mathrm{PD}}_{\left(x,y,z\right)}}{dt}=\frac{\varphi_{\left(x,y,z\right)}\times \mathrm{ETE}}{E_{540}}\times \gamma ,$$ (1)

where ETE is the overall energy transfer efficiency, calculated as ETE = ET1 × ET2 × ET3, $\varphi_{\left(x,y,z\right)}$ is the local fluence in the voxel, $E_{540}$ is the energy per photon at 540 nm, and γ is the quantum yield of the singlet oxygen produced by the photosensitizer. Briefly, the fluence absorbed by UCQRs in each voxel is converted to that absorbed by RB by multiplying it by the ETE. This fluence is then converted to the number of photons by dividing by the energy per photon at 540 nm. Finally, by multiplying by the quantum yield, the number of excitation photons that contribute to singlet oxygen generation by RB is obtained. Based on previous studies, we assumed that the oxygen concentration within the tumor was always sufficient for generating singlet oxygen.^39^ In this study, we assumed that tumor cell death occurs only when the PD exceeds a threshold dose, as previously reported by Farrell et al.^38^ This assumption is based on observations in some PDT studies, which showed a distinct boundary between cell death and non-cell death tissue regions.^38^ In the simulations, voxels in which the PD exceeded the threshold value of $8.6\times 10^{17}\;\text{photons}/{\mathrm{cm}}^{3}$, which has been established as the cell death threshold for skin cancer, following one or multiple irradiation sessions, were considered to have undergone cell death.^39^

#### 2.3.4 Simulation settings

To estimate the therapeutic efficacy of NIR-PDT using UCQRs, MC simulations were performed for light irradiation at an excitation wavelength of 808 nm, which is the wavelength of UCQRs. As shown in Table 1, each voxel in the phantom was assigned the optical properties of the tissue at 808 nm, which were determined based on previously reported values, as well as factors such as the volume ratio of glandular tissue to fat.^40^^–^^43^ The detailed methodology for setting these parameters is described in previous studies.^22^ Here, the light propagation at 410, 660, and 1540 nm emitted from Er was not analyzed because it does not contribute to the excitation of RB and thus does not influence the PDT efficacy. Furthermore, the 540-nm emission from Er was also excluded from the simulation, as its energy is transferred to the RB via RET without propagating through the tissue. The concentration of UCQRs within the tumor was set to 75  mg/kg, based on the tumor concentrations observed in mouse experiments.^16^ In addition, the absorption coefficient of UCQRs in the tumor was set to $0.38\;{\mathrm{cm}}^{-1}$, which was calculated from the UCQR concentration and the molar absorptivity of QDs.^16^ This absorption coefficient was then added to that of the corresponding tumor voxel.

**Table 1:** Optical properties of the breast tissue at a wavelength of 808 nm.

Tissue	$\mu_{a}\;$^a^ (${\mathrm{cm}}^{-1}$)	$\mu_{s}\;$^b^ (${\mathrm{cm}}^{-1}$)	n ^c^	g ^d^
Skin	1.35	196	1.37	0.9
Fat	0.09	108	1.45	0.9
Milk duct	0.06	114	1.42	0.9
Tumor with UCQRs	0.48	98	1.45	0.9

a Absorption coefficient

b Scattering coefficient

c Refractive index

d Anisotropy

In this study, the irradiation intensity was set to $330\;\mathrm{mW}/{\mathrm{cm}}^{2}$, which falls within the maximum permissible exposure for skin at 808 nm, as defined by the American National Standard for Safe Use of Lasers.^44^ For reference, in PDT for skin cancers, a typical total treatment dose for a single light exposure in the 630 to 690 nm range is $200\;J/{\mathrm{cm}}^{2}$.^45^ In the simulation, the irradiation intensity and duration for a single exposure were $330\;\mathrm{mW}/{\mathrm{cm}}^{2}$ and 606 s, respectively. The excitation light was directed parallel to the body axis. Multiple light irradiations were assumed in this study to achieve a 100% therapeutic effect of NIR-PDT with UCQRs on the tumor.

## 3 Results and Discussion

### 3.1 Classification of the Breast Phantoms

**Fig. 3:**
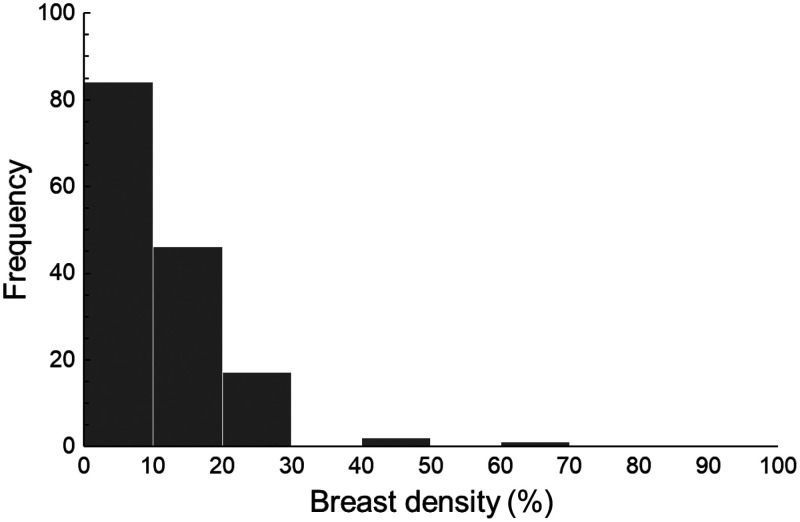
Histogram showing the breast density in 150 phantoms. As shown in the histogram, the breast density of most phantoms, obtained from BreastCT, was below 30%.

The distribution of breast density in the 150 phantoms was statistically analyzed (Fig. 3). The breast density was below 10% in 84 phantoms, 10% to 20% in 46 phantoms, and 20% to 30% in 17 phantoms. Only three phantoms exhibited a breast density over 30%, and none exceeded 70%. According to the BI-RADS classification, breasts with a density of 0 to 25% are categorized as fatty, 25% to 50% as scattered glandular, 50% to 75% as heterogeneously dense, and 75% to 100% as extremely dense. Based on this classification, 135 out of 150 phantoms (90%) were identified as fatty breasts. Ten (7%) and five (4%) phantoms were categorized as scattered glandular and heterogeneously dense, respectively, whereas none was classified as extremely dense. In a previous study, the breast density of 200 women was evaluated using mammographic images. Quantitative analysis showed that 105 women (53%) had fatty breasts.^46^ Thus, among the 150 BreastCT phantoms, the proportion classified as fatty breasts (90%) was notably high.

Although BI-RADS is primarily used for breast images from mammography, it has been noted to overestimate breast density. Moreover, the average breast density in mammography is typically assumed to be 50%, whereas the BreastCT data from 191 women showed a lower value of 19.3%.^47^ Therefore, in this study, the breast density of the 150 phantoms obtained from BreastCT was multiplied by 2.5, and these adjusted breast densities were used to reclassify the 150 phantoms using the BI-RADS criteria (Table 2). Groups comprising 87 (58%), 43 (29%), 17 (11%), and 3 phantoms (2%) were classified as fatty breasts, scattered fibroglandular, heterogeneously dense, and highly dense breasts, respectively. Because DCIS occurs exclusively within the ductal system, it is unlikely to develop in breasts with a density of 10% or less. Consequently, the 87 phantoms classified as fatty breasts were excluded from the estimation of therapeutic effects using MC simulations. In addition, the 24 phantoms that were unable to accommodate tumors in the typical location due to insufficient space within the ductal structure were also excluded.

**Table 2:** Classification of the 150 phantoms based on the newly established criteria.

	Number of phantoms	Percentage (%)
Fatty breast	87	58
Scattered fibroglandular breast	43	29
Heterogeneously dense breast	17	11
Extremely dense breast	3	2

**Fig. 4:**
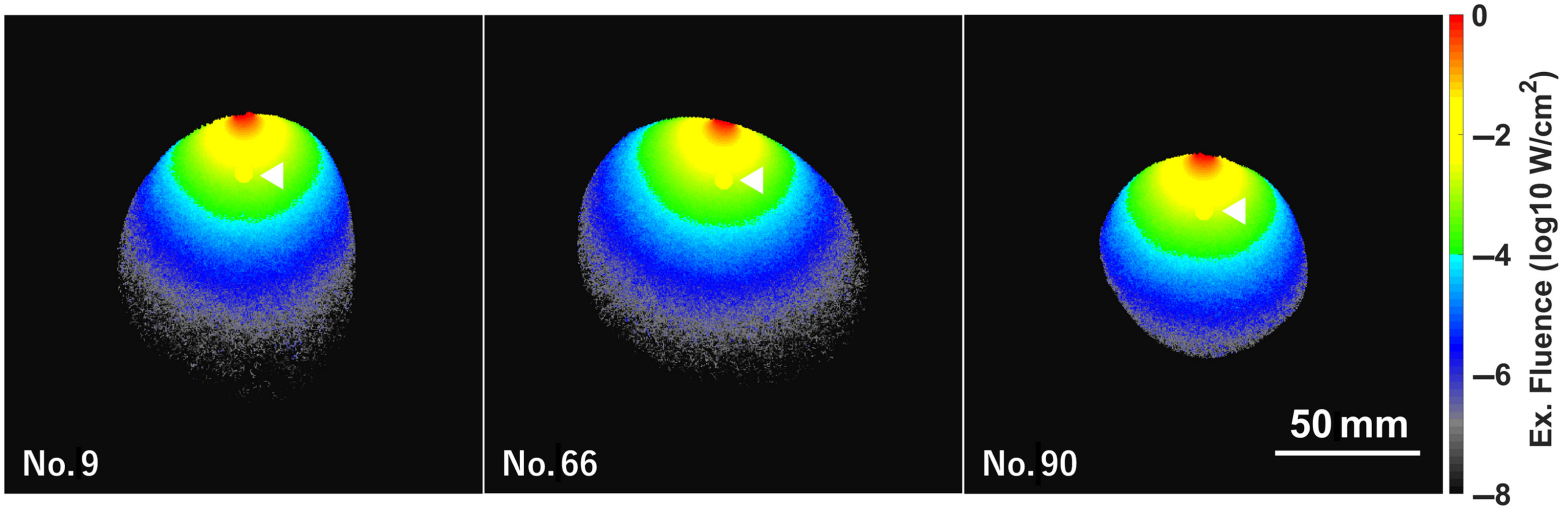
Typical color maps of the excitation fluence at 808 nm in phantom nos. 9, 66, and 90. Each image is a cross-sectional slice at the tumor center. In all models, the tumors had a diameter of 7 mm and a depth of 20 mm. The breast densities of phantom nos. 9, 66, and 90 are 40, 58, and 32%, respectively. In other words, phantom nos. 9 and 66 are classified as scattered fibroglandular breasts, and phantom no. 90 is classified as a heterogeneously dense breast in Table 2. The excitation light was irradiated from above the tumor. The intensity of the irradiating light was $330\;\mathrm{mW}/{\mathrm{cm}}^{2}$, as indicated in Sec. 2. The white scale bar represents 50 mm. The white arrowhead indicates the fluence distribution within the 7-mm-diameter tumor. The optical properties of all phantoms are given in Table 1.

In 39 of the remaining phantoms, the tumors were embedded at appropriate locations within the mammary gland based on four specified criteria. The influence of the structure and contour of the mammary gland on the therapeutic effect was then investigated. Figure 4 presents the results of the excitation light propagation at 808 nm for patient nos. 9, 66, and 90. These phantoms were randomly selected from a group with 7-mm-wide tumors located at a depth of 20 mm, corresponding to the midpoint of the common range (15 to 25 mm). The cross-sectional shape, size, and light propagation depicted in the fluence map varied according to the contour and internal structure of each phantom. In the tumor voxels, the absorption of excitation light was enhanced by UCQRs, resulting in higher fluence levels compared with the surrounding voxels. The fluences in spherical tumors (7 mm in diameter at 20-mm depth) were comparable across the samples. The average tumor fluence was $18.3\;\mathrm{mW}/{\mathrm{cm}}^{2}$ for no. 9, 19.5 for no. 66, and 23.2 for no. 90. The difference between the highest and lowest values was $4.9\;\mathrm{mW}/{\mathrm{cm}}^{2}$. For the tumor in o. 9, the average fluence values at depths of 19, 20, and 21 mm were 29.9, 18.3, and $10.0\;\mathrm{mW}/{\mathrm{cm}}^{2}$, respectively. The average fluence difference between 19 and 20 mm ($11.6\;\mathrm{mW}/{\mathrm{cm}}^{2}$) and 20 to 21 mm ($8.3\;\mathrm{mW}/{\mathrm{cm}}^{2}$) was $10.0\;\mathrm{mW}/{\mathrm{cm}}^{2}$. For no. 66, the respective differences were 12.4 and $9.3\;\mathrm{mW}/{\mathrm{cm}}^{2}$, with an average of $10.9\;\mathrm{mW}/{\mathrm{cm}}^{2}$; for no. 90, the differences were 12.6 and $7.9\;\mathrm{mW}/{\mathrm{cm}}^{2}$, with an average of $10.3\;\mathrm{mW}/{\mathrm{cm}}^{2}$. Thus, the difference in the mean fluence within the tumor due to a change in the tumor depth (1 mm) is more than twice that due to the phantom of each patient.

Therefore, in this study, phantoms with tumors at the same depth were assumed to exhibit similar therapeutic effects. Table 3 presents the results of classifying 39 phantoms based on tumor depth, with most of them (19 phantoms) grouped at a tumor depth of 15 mm, which is attributed to criterion (4). In addition, no phantoms were classified in the 24-mm depth group, as no phantom was capable of completely embedding a tumor at that depth. MC simulations were conducted on phantoms with the average mammary gland density within each group.

**Table 3:** Number of phantoms in which a tumor could be embedded at each depth from 15 to 25 mm.

Tumor depth (mm)	15	16	17	18	19	20
Number of phantoms	19	1	1	2	1	5
	21	22	23	24	25	—
	4	2	2	0	2	—

### 3.2 Assessment of Therapeutic Effects of NIR-PDT

**Fig. 5:**
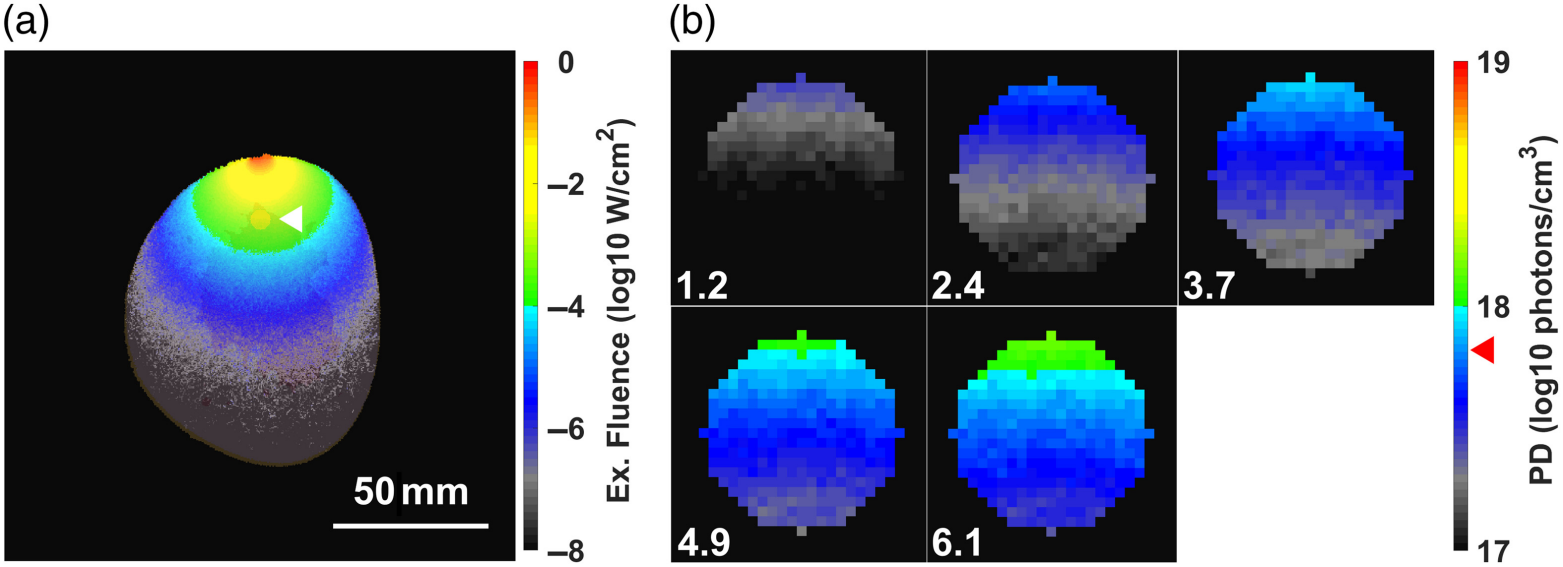
Color maps of excitation fluence and singlet oxygen (O12) generation by UCQRs for each ETE in phantom no. 9. (a) Cross-section of the tumor center of the phantom. The fluence map is overlaid with the internal breast structure. The scale bar (50 mm) is shown by a white line, and the white arrowhead denotes the 7-mm-diameter tumor. (b) Plotted singlet oxygen generation represents the total amount produced during a single session (606 s). The ETE values for only the tumor voxels in the cross-section are shown in the lower-left corner of each panel. In the PD color bar, the red arrowhead indicates the apoptosis threshold ($8.6\times 10^{17}\;\text{photons}/{\mathrm{cm}}^{3}$).

Figure 5 shows the excitation fluence map at 808 nm (a) and the color maps of the PD within the tumor at ETE levels ranging from 1.2% to 6.1% (b) in phantom no. 9, one of five phantoms with a tumor depth of 20 mm. The total PD in the tumor voxels at each ETE level was 0.256, 0.514, 0.767, 1.02, and $1.28\times 10^{18}\;\text{photons}/{\mathrm{cm}}^{3}$ [Fig. 5(b)], indicating a direct correlation between the ETE and the total PD in the tumor. However, as indicated by the PD threshold in the figure, the amount of singlet oxygen generated in the deeper tumor regions did not surpass the threshold with a single light irradiation.

**Fig. 6:**
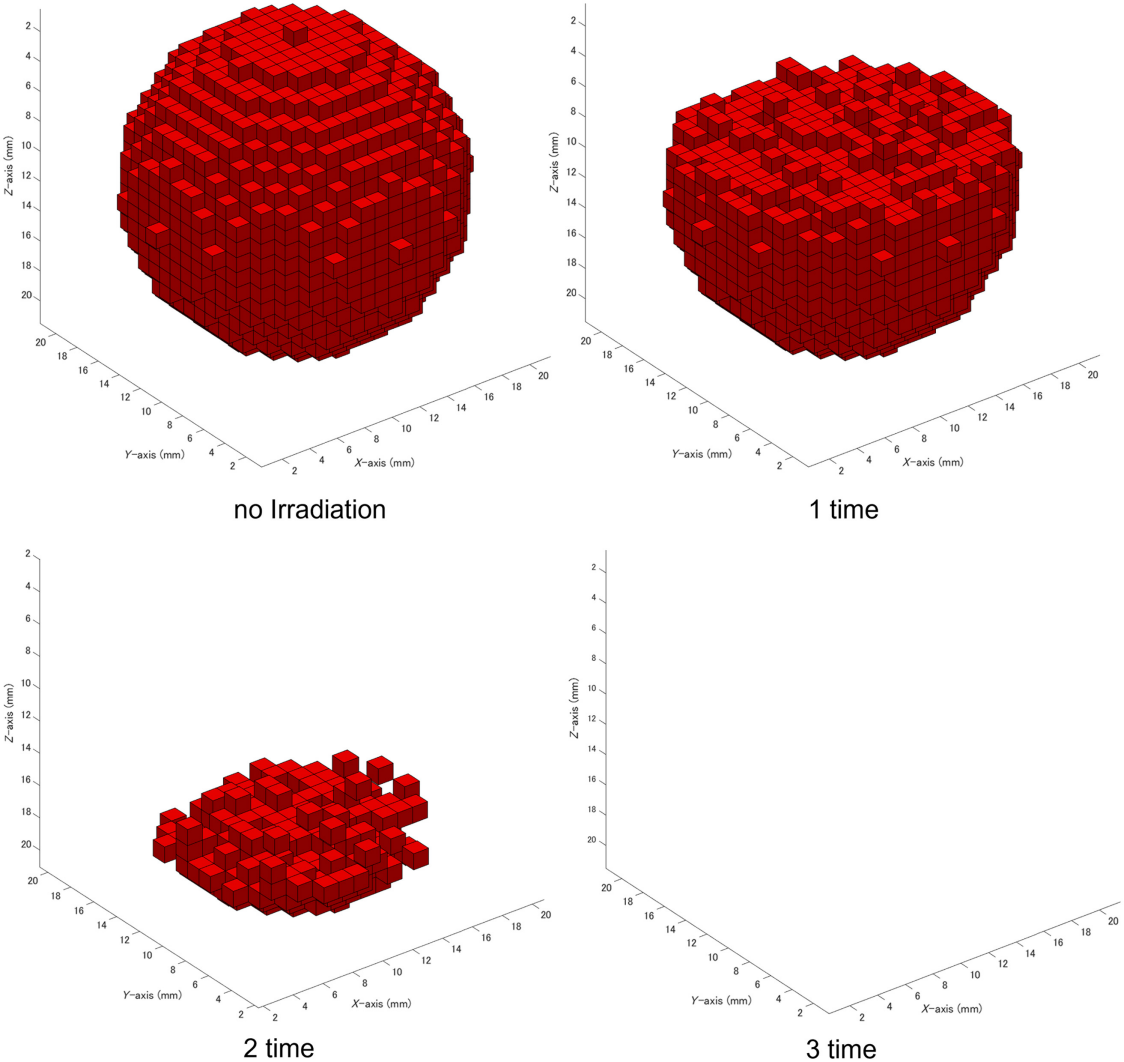
3D representation of the tumor after each irradiation in phantom no. 9. The number of light irradiations is indicated below each panel. The tumor was completely treated after three irradiations. The x-, y-, and z-axes represent the width, length, and height of the tumor, respectively. Note that the z-axis does not represent the tumor depth.

Next, the number of light irradiations required to fully treat the tumor was evaluated. Figure 6 illustrates the 3D representation of the tumor after zero to three irradiations in phantom no. 9 with a tumor depth of 20 mm. As the number of irradiations increased, singlet oxygen above the threshold was produced in the tumor voxels closer to the skin surface. Consequently, only the upper tumor voxels were treated initially, whereas the lower ones remained unaffected. After three light irradiations, all tumor voxels were successfully treated.

**Fig. 7:**
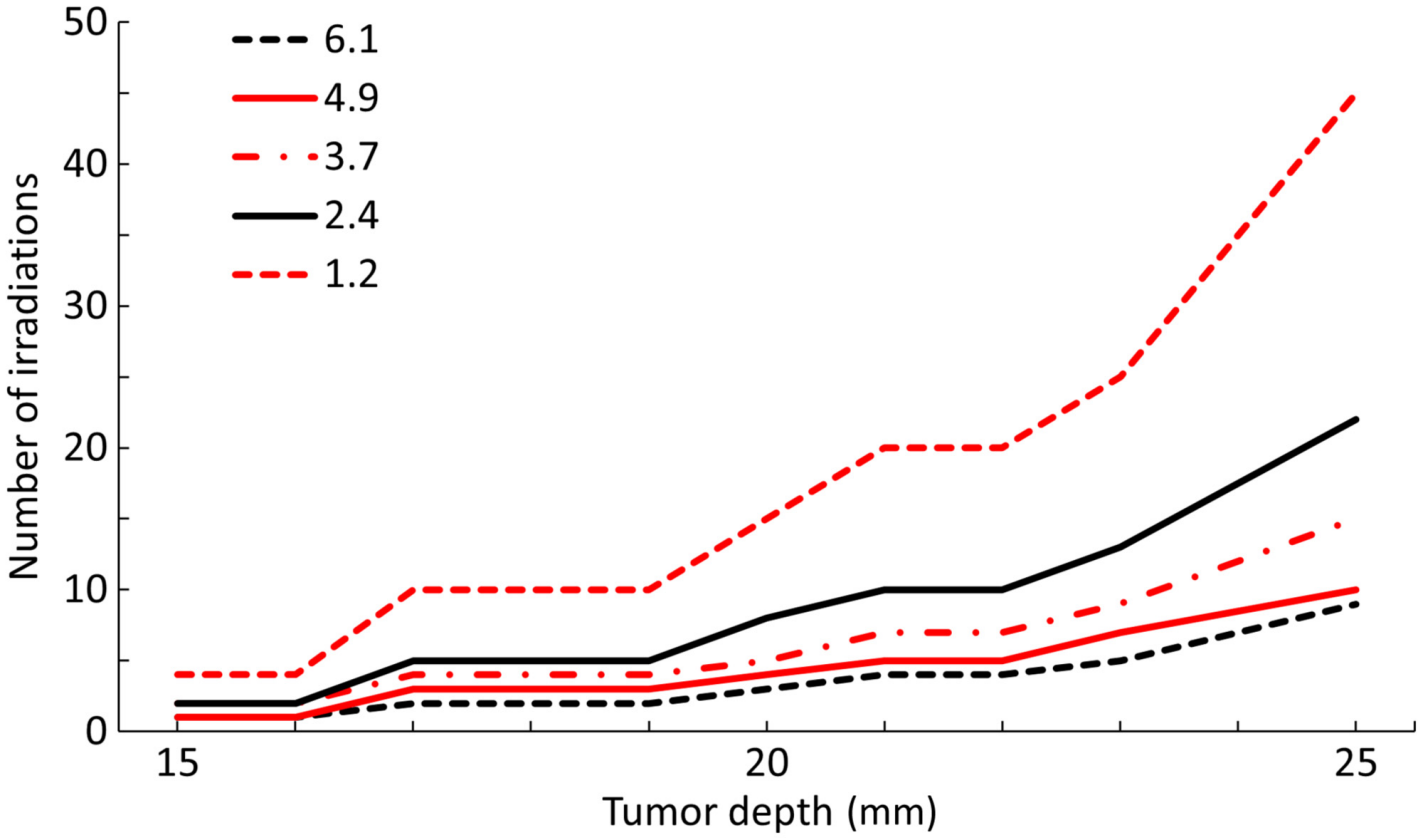
Number of irradiations required to completely treat tumors at each tumor depth for ETEs ranging from 1.2% to 6.1%. The x- and y-axes represent the tumor depth in the phantom and the number of irradiations needed for complete tumor treatment, respectively.

Figure 7 illustrates the number of irradiations required to completely treat tumors with a 7-mm diameter at various tumor depths for ETEs ranging from 1.2% to 6.1%. At an ETE of 1.2%, 4 irradiations were needed for a tumor depth of 15 mm, whereas 45 irradiations were required for tumors at a depth of 25 mm. In contrast, at an ETE of 6.1%, only one irradiation was sufficient for tumors at a depth of 15 mm, whereas only nine were required for those at a depth of 25 mm. Thus, the number of irradiations decreased with increasing ETE, and this effect was more pronounced for tumors at higher depths.

In general, excitation lifetimes are extremely short (nanoseconds or picoseconds), making two-photon excitation unlikely. However, due to the relatively long excitation lifetimes of trivalent lanthanide ions such as Er,^37^ when incorporated into host lattices such as NaYF4 as upconversion nanoparticles, these lifetimes are further extended.^37^ We set the probability of two-photon excitation in Yb-Er to 20% to 100%. Also, among the four energy transfer pathways in Yb-Er, we set the energy transfer efficiency to 540 nm, which matches the excitation of RB, to 50%.^22^ Therefore, by multiplying these, the ET2 at 540 nm is calculated to be 10% to 50%. Then, because the lanthanide ions (Er) incorporated into the host lattice have a long excitation lifetime (>10  ms), we expected the ET2 to be close to the maximum of 50%. However, Fischer et al.^48^ reported that the energy transfer efficiency between Yb and Er incorporated in β-NaYF4 was 2.0% at 1523 nm. As the energy transfer efficiency in the pathway around 1540 nm is about one-third of the efficiency at 540 nm, the ET2 at 540 nm would likely be at least 6.0% higher.^22^ Furthermore, in UCQR, the absorption of QDs on UCNPs enhances the absorption of excitation light, and experiments have reported that the luminescence intensity of UCNPs increased 20-fold by QDs. Therefore, the energy transfer efficiency of Yb-Er in UCNPs with QDs would be significantly higher than that in UCNPs alone. Therefore, 50% ET2 may be an overestimation, but 10% to 20% would be a realistic range. In other words, the overall ETE for UCQR will be close to 1.2% to 2.4%. Therefore, Fig. 7 suggests that tumors located at shallow depths (15 to 20 mm) within the typical range can be completely treated by NIR-PDT using UCQRs with fewer than 15 light irradiations at the ETE of 1.2%. In tumors located at deeper depths (20 to 25 mm) within the typical range, up to 20 irradiations are required at the ETE of 2.4%, whereas as many as 45 irradiations are needed at the ETE of 1.2%. On the other hand, tumors within the all depth range can be treated entirely by NIR-PDT using UCQRs at an ETE of 6.1% with fewer than 10 light irradiations. Therefore, the development of nanoparticles with higher upconversion efficiency for NIR-PDT is desirable.

### 3.3 Generation of Singlet Oxygen in the NIR-PDT

This study assumed sufficient oxygen to be present within tumors at all times, based on the Monte Carlo simulation study of skin cancer by Valentine et al.^39^ However, in chronically hypoxic *in vivo* tumors, the amount of singlet oxygen generated by PS is limited, which tends to reduce the therapeutic efficacy of PDT.^49^ In a previous study, Penjweini et al.^50^ estimated the therapeutic efficacy of PDT by simulation, considering the oxygen consumption by PS. They reported that the amount of singlet oxygen generated is strongly influenced by the initial oxygen concentration within the tumor and that the effect is stronger when the initial oxygen concentration is below 10  μM. In breast cancer tumors, the amount of singlet oxygen generated varies because local concentrations can be significantly below 10  μM.^51^ Therefore, the amount of singlet oxygen generated calculated from our simulation may be overestimated, which is a limitation of the simulation in this study. Meanwhile, Finlayson et al.^29^ conducted a simulation of PDT for glioblastoma to investigate the effect of intratumoral oxygen depletion on PDT treatment efficacy. Their results indicated that there was little difference in the survival rate at the end of treatment, regardless of whether oxygen depletion occurred or not. This is thought to be because the oxygen recovery rate in brain tissue is significantly higher than the PDT oxygen utilization rate, allowing oxygen levels to recover quickly enough.^29^ However, readers should note that simple comparisons cannot be made due to the differences in the types of cancer tumors and PSs targeted. Given these factors, simulations that take into account differences in initial oxygen concentration, oxygen recovery rate, and local oxygen concentration within the tumor are an important topic for future study.

### 3.4 Monte Carlo Simulation in the NIR-PDT

We assumed that early-stage breast cancer tumors without metastases were the target of NIR-PDT treatment. A previous MC simulation study conducted on tumors with diameters ranging from 5 to 9 mm suggested that tumors <1  cm in diameter, occurring within a common range of 15 to 25 mm from the skin, could be completely treated with fewer than 10 light irradiations. The number of irradiations required for treatment was also estimated to increase with increasing tumor size in NIR-PDT. Therefore, early detection of breast cancer is crucial to ensure effective therapy by NIR-PDT. Mammography, which is currently used to diagnose breast cancer, has a screening sensitivity of over 90% in breast cancer tumors within 10 mm in diameter.^52^ Furthermore, MRI shows a screening sensitivity of more than 90% in DCIS.^53^ Therefore, tumors with a diameter of 7 mm, used in this study, are large enough to be detected by current diagnostic methods, suggesting that NIR-PDT is an effective treatment for realistically detectable early-stage breast cancer tumors.

In this simulation model, the tumor shape was limited to spherical, and its effect on treatment efficacy was not investigated. Studies utilizing three-dimensional models that accurately replicate actual tumor geometries are currently limited. Moreover, to the best of our knowledge, there are no publicly available datasets that provide such 3D tumor shape information.^23^ As evident from the excitation fluence maps in this study, the delivery of light energy to the tumor is strongly influenced by the distance from the irradiation point. Due to the complexity of the tumor geometry, the production of singlet oxygen within the tumor may not reach the therapeutic threshold. Therefore, reproducing the actual tumor geometry in PDT simulations contributes to a more accurate estimation of the therapeutic effect. Hence, future studies should incorporate 3D models that faithfully reproduce the actual tumor geometry.

The results of the MC simulations are strongly influenced by the optical properties. As the scattering coefficient increases, light penetration into the tissue is limited, and the light energy reaching the tumor is weaker. In addition, as the absorption coefficient of the tissue increases, a significant portion of the energy is absorbed by the biological tissue before the light reaches the PS in the tumor, compromising the therapeutic effect of PDT. In this study, the optical properties of breast tissue at 808 nm were established using the equation for generating the optical properties of typical tissue at any wavelength reported in previous studies.^20^ This equation was generated based on the variable amounts of absorbing chromophores (blood, water, melanin, fat, and yellow pigment) and the variable balance between small-scale and large-scale scatterers in the ultrastructure of cells and tissues. However, the absorption coefficients were estimated for the breast as a whole, but not separately for the mammary gland and fat. We assumed that the absorption coefficient of fat was the same as that of the whole breast, as most of the breast tissue consists of fat. The absorption coefficient of the mammary gland was then obtained by multiplying the absorption coefficient of fat by the ratio of the absorption coefficient of the mammary gland and fat reported in another study (0.56).^22^ Furthermore, the optical properties of the skin tissue were obtained by considering the ratio of dermis to epidermis thickness in the breast. Therefore, the voxels of the simulation phantom were assigned appropriate optical properties for each tissue, enabling the MC simulation on a phantom that closely mimics light propagation in the actual breast. The exact optical properties of mammary glands and fat should be investigated to enable a more rigorous simulation.

## 4 Conclusion

In this study, the therapeutic effect of NIR-PDT for breast cancer was evaluated using 150 breast phantoms through MC simulations. The results indicated that the efficacy of NIR-PDT was influenced more by tumor depth than by mammary structure or breast shape. Furthermore, for tumor depths within the standard range of 15 to 25 mm, complete treatment was achieved through multiple irradiations, regardless of the specific depth or ETE. In 1.2% to 2.4% of ETE cases, shallow tumors (15 to 20 mm) were treated with 15 or fewer irradiations, whereas deep tumors (20 to 25 mm) were estimated to require up to 45 irradiations. In addition, at a maximum ETE of 6.1%, all tumor-depth phantoms were estimated to be treatable with fewer than 10 irradiations. Therefore, these findings support the effectiveness of NIR-PDT using UCQRs as a promising treatment approach for breast cancer. It was also suggested that more efficient upconversion nanoparticles would significantly improve the therapeutic efficacy of NIR-PDT.

## Data Availability

The data underlying the results presented in this paper are not publicly available at this time but may be obtained from the authors upon reasonable request.
